# The number of episodes of major psychiatric and substance use disorders as an index of genetic risk and genetic heterogeneity

**DOI:** 10.1038/s41380-024-02727-x

**Published:** 2024-09-02

**Authors:** Kenneth S. Kendler, Henrik Ohlsson, Jan Sundquist, Kristina Sundquist

**Affiliations:** 1 Virginia Institute for Psychiatric and Behavioral Genetics, Virginia Commonwealth University, Richmond, VA USA; 2 Department of Psychiatry, Virginia Commonwealth University, Richmond, VA USA; 3 Center for Primary Health Care Research, Department of Clinical Sciences, Lund University, Malmö, Sweden; 4 University Clinic Primary Care Skåne, Region Skåne, Sweden

**Keywords:** Genetics, Psychiatric disorders

## Abstract

We investigate whether number of episodes (NoEs) meaningfully reflect genetic risk and genetic heterogeneity for five primary disorders—Drug Use Disorder (DUD), Alcohol Use Disorder (AUD), Major Depression (MD), Bipolar Disorder (BD), and Schizophrenia (SZ) ascertained from Swedish population registries. We utilize Genetic Risk Ratios (GRR)—defined as the ratio of the genetic risk for secondary disorders to the genetic risk for the primary disorder—derived from Family Genetic Risk Scores (FGRS). For all five primary disorders, genetic risk rose robustly with increasing NoEs. For both AUD and DUD, the GRR for all six secondary disorders—selected to have a likely genetic relationship with the particular primary disorder—declined with increasing NoEs so that cases of AUD and DUD with high versus low NoEs had both a higher genetic risk and a purer genetic signal. With MD, genetic risk maximized at an intermediate NoEs. While the GRRs for AUD and DUD in MD cases dropped sharply with increasing NoEs, GRR for BD increased. For BD, genetic risk rose sharply with increasing NoEs while for all secondary disorders the GRRs showed a mixture of modest increases and decreases. Like AUD and DUD, but even more markedly, selecting BD cases with high rates of recurrence would produce a sample with a high overall genetic risk and a relatively homogeneous genetic signal. For SZ, genetic risk rose moderately with increases in NoEs. GRRs for other non-affective psychoses (ONAP) and autism spectrum disorder (ASD) fell quite slowly with increasing NoEs, and more rapidly for other secondary disorders. Cases of SZ with high recurrence rates had a high genetic risk and a relatively pure signal, albeit with contributions from ONAP and ASD. In summary, NOEs are a robust index of genetic risk and genetic heterogeneity across our primary disorders with important inter-disorder differences.

The number of episodes (NoEs) in major psychiatric and substance use disorders is a central feature of illness course, with high rates often associated with poor clinical outcome and increased social impairment [1–3]. While many studies have looked at clinical predictors of NoEs across disorders [4–9], only a few have included genetic/familial risk factors. These studies have found that genetic risk tends to positively predict NoEs in major depression (MD) [10–13], alcohol use disorder (AUD) [14], and drug use disorder (DUD) [8, 12].

In this report, using large Swedish national samples of five primary disorders (AUD, DUD, MD, bipolar disorder (BD), and schizophrenia (SZ)), we begin by examining the relationship between genetic risk—calculated by the Family Genetic Risk Score (FGRS) [15–18]—and NoEs. We then expand our consideration of NoEs to determine whether they index genetic heterogeneity in these disorders.

To do this, we defined a novel genetic construct termed “Genetic Relative Risk” (GRR). For a particular primary disorder with a given NoEs, the GRR equals the ratio of the genetic risk for secondary disorders to the genetic risk for the primary disorder. Secondary disorders were selected because of a likely genetic relationship with the particular primary disorder. Assume, for example, that we are measuring the genetic risk on a standardized scale for cases of MD, our primary disorder, who have had 5 episodes of illness over a set period of time. This equals 0.30. For those same MD cases, they have, on the same standardized scale, a genetic risk for BD of 0.15. Then, for cases of MD with 5 episodes, the GRR for BD equals 0.15/0.30 or 0.50.

We calculate GRRs for each number of episodes, varying from 1–12 to 1–26, across our five primary psychiatric and substance use disorders. We then evaluate whether the NoEs is a meaningful index of genetic heterogeneity by examining whether the GRRs for 6–8 relevant secondary psychiatric disorders change meaningfully as a function of the NoE for these primary disorders.

## Methods

We collected information on individuals from Swedish population-based registers with national coverage linking each person’s unique personal identification number which, to preserve confidentiality, was replaced with a serial number by Statistics Sweden. We secured ethical approval for this study from the Regional Ethical Review Board in Lund and no participant consent was required (No. 2008/409 and later amendments).

**Fig. 1:**
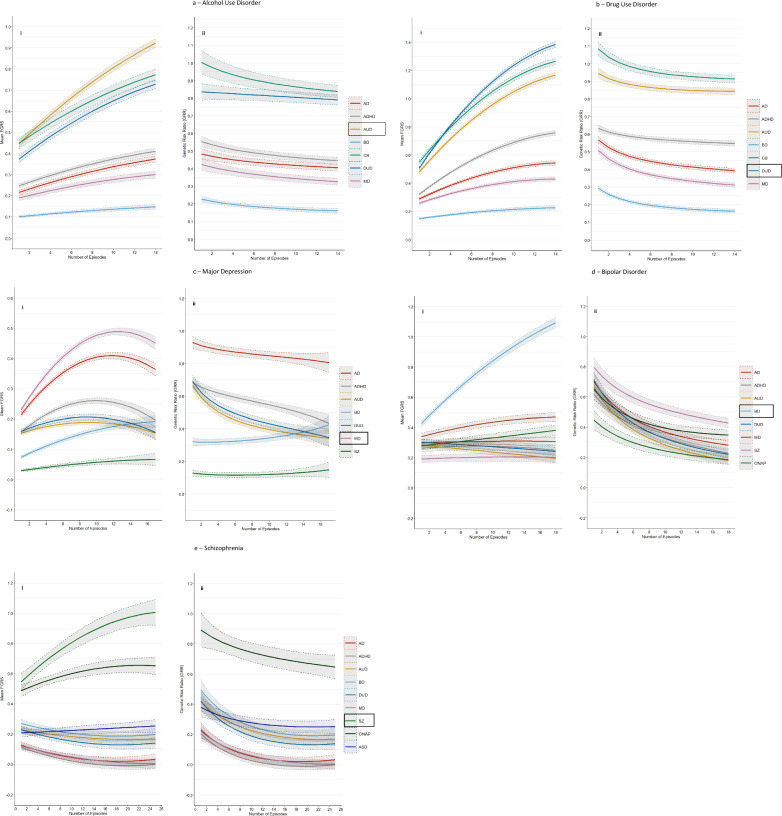
Mean Family Genetic Risk Score and Genetic Risk Ratio results for Alcohol Use Disorder, Drug Use Disorder, Major Depression, Bipolar Disorder and Schizophrenia. **a** Mean Family Genetic Risk Score (FGRS) (part i) and Genetic Risk Ratio (part ii), both ± 95% CIs, in individuals with Alcohol Use Disorder for number of episodes from 1 to 14. Part i includes the primary disorder (here Alcohol Use Disorder) with darkened 95% CIs, and the secondary disorders/traits (here Anxiety Disorder (AD), ADHD, Bipolar Disorder (BD), Criminal Behavior (CB), Drug Use Disorder (DUD), and Major Depression (MD)). Part ii only includes the secondary disorders/traits. **b** Mean Family Genetic Risk Score (FGRS) (part i) and Genetic Risk Ratio (part ii), both ± 95% CIs, in individuals with Drug Use Disorder for number of episodes from 1 to 14. Part i includes the primary disorder (here Drug Use Disorder), with darkened 95% CIs, and the secondary disorders/traits (here Anxiety Disorder (AD)), ADHD, Bipolar Disorder (BD), Criminal Behavior (CB), Alcohol Use Disorder (DUD), and Major Depression (MD)). Part ii only includes the secondary disorders/traits. **c** Mean Family Genetic Risk Score (FGRS) (part i) and Genetic Risk Ratio (part ii), both ± 95% CIs, in individuals with Major Depression for number of episodes from 1 to 16. Part i includes the primary disorder (here Major Depression), with darkened 95% CIs, and the secondary disorders/traits (here Anxiety Disorder (AD), ADHD, Alcohol Use Disorder (AUD, Bipolar Disorder (BD), Drug Use Disorder (DUD), Major Depression (MD) and Schizophrenia). Part ii only includes the secondary disorders/traits. **d** Mean Family Genetic Risk Score (FGRS) (part i) and Genetic Risk Ratio (part ii), both ± 95% CIs, in individuals with Bipolar Disorder for number of episodes from 1 to 18. Part i includes the primary disorder (here Bipolar Disorder), with darkened 95% CIs, and the secondary disorders/traits (here Anxiety Disorder (AD), ADHD, Alcohol Use Disorder (AUD), Drug Use Disorder (DUD, Major Depression (MD), Schizophrenia and Other Non-Affective Psychosis (ONAP)). **e** Mean Family Genetic Risk Score (FGRS) (part i) and Genetic Risk Ratio (part ii), both ± 95% CIs. in individuals with Schizophrenia for number of episodes from 1 to 25. Part i includes the primary disorder (here Schizophrenia (SZ), with darkened 95% CIs, and the secondary disorders/traits (here Anxiety Disorder (AD), ADHD, Alcohol Use Disorder (AUD), Bipolar Disorder (BD), Drug Use Disorder (DUD), Major Depression (MD), Other Non-Affective Psychosis (ONAP) and Autism Spectrum Disorder (AUD)).

We begin by creating five separate datasets based on individuals registered for our five primary disorders and calculate the number of episodes (NoEs) for each individual. In order to be counted as a new episode, the registration in the dataset had to occur at least 90 days after the prior registration. Individuals were born between 1940–2003 in Sweden to Swedish born parents. For details of the relevant registers, which includes information from national patient and primary care registers, and diagnostic codes, see appendix Tables 1 and 2 and Fig. 1, respectively. In the datasets (details in appendix Tables 3), we also included individual family genetic risk scores (FGRS) for primary and secondary disorders. FGRSs are calculated from morbidity risks for disorders in first- through fifth-degree relatives, controlling for cohabitation effects, and thus arise from phenotypes in extended pedigrees, not from molecular genetic data. For details, see appendix Table 4. In the databases, we also included year of birth, age at first registration for the disorder and follow-up time calculated as number of years from first registration to end of follow-up (2018-12-31, date of death, date of emigration whichever came first).

To examine FGRS profiles for different NoEs, we first calculated the predicted NoE using a linear regression model with NoE as outcome and year of birth, age at first registration and time of follow-up as predictor variables. Then we used a multilevel linear regression model with the FGRSs as outcome and NoE as predictor variables, including a linear and quadratic term for NoE. To account for variable length of follow-up, year of birth and age at registration, we included a separate stratum for each percentile of the predicted NoE (e.g., individuals with similar predicted NoE belong to the same stratum). This means that we are investigating the association between the actual value of NoE and FGRSs among individuals with similar values of predicted NoE. We present, in our primary disorders, the predicted FGRS for NoEs up to the 99th percentile of the empirical distribution. Thereafter, we calculated GRRs for each NoE, where the denominator was the FGRS for the primary disorder, and the numerator was the FGRSs for the secondary disorders or traits. A core of 6–8 secondary disorders/traits were examined for all disorders (AD [Anxiety Disorders], ADHD, AUD, BD, DUD, and MD) but others were chosen a priori for particular primary individual disorders for their potential importance (e.g, criminal behavior (CB) for AUD and DUD and Autism Spectrum Disorder (ASD) for SZ). We also present the linear slope of all GRRs and all FGRSs across the NoEs. All analyses were also presented separated by sex. In two sensitivity analyses, we present the GRRs based on two separate birth cohorts (1955-64 and 1965-74), and by excluding individuals with relevant comorbidities (see appendix Tables 5 and 6). In a final sensitivity analysis, we compared the pattern of results for the first 5-year period after the initial registration with the second 5-year period. All statistical analyses were performed using SAS 9.4 [19] and/or R 4.3.1 [20].

## Results

Table 1 depicts the sample size, sex distribution and mean NoEs for our five primary disorders. Sample sizes ranged from 25,646 cases of SZ to 792,247 cases of AD. BD, MD and AD had the expected female excess and DUD, AUD, and SZ the expected male excess. The distribution of the NoEs for our five disorders are seen in appendix Fig. 2a–e. The distributions are all “one-inflated” with long rightward tails, with the percent of individuals with a single episode ranging from 23% for SZ to 50% for DUD.

**Table 1:** Number of Cases of Identified from the Swedish Population born 1940–2003 for Drug Use Disorder (DUD), Alcohol Use Disorder (AUD), Major Depression (MD), Bipolar Disorder (BD), and Schizophrenia (SZ).

	DUD	AUD	SZ	BD	MD^a^
*N*	208,030	304,062	25,646	72,057	792,247
Year of Birth (Mean, SD)	1976 (16.5)	1961 (15.1)	1959 (13.2)	1969 (16.6)	1969 (16.7)
Males (%)	68.2%	72.5%	60.5%	39.0%	37.1%
Mean number of episodes (Mean, SD)	3.2 (4.0)	3.8 (4.6)	8.3 (7.9)	5.3 (5.6)	2.8 (2.7)
FGRS (Mean, SD)	0.680 (1.6)	0.542 (1.4)	0.730 (2.8)	0.616 (1.9)	0.286 (1.1)
Mean year of follow-up (Mean, SD)	10.6 (8.6)	16.4 (12.8)	19.2 (12.9)	10.7 (9.9)	7.5 (5.4)

^a^All BD cases are excluded.

The main analyses of our five primary disorders are presented in Fig. 1a–f. (For individual values, see appendix Tables 7a–e). The left hand figure (i) presents results for the mean FGRS (±95% CIs) for the relevant primary disorder and for our secondary disorders *in cases with the primary disorder* as a function of their NoEs. The right hand figure (ii) takes these results and transforms them into the GRR (±95% CIs) for each of the secondary disorders. The GRR captures the relative change, across number of episodes, in the genetic risk for the secondary disorder and the primary disorder. The “null hypothesis”—that the GRR is not influenced by the NoEs—that is, that the genetic relationship between the primary and secondary disorder is constant over the level of recurrence, would result in a flat GRR, a result very rarely seen in Fig. 1aii to 1fii. In almost every instance, the GRRs for the secondary disorders change as a function of the NoEs of the primary disorder. We describe our findings in detail for the first primary disorder—AUD.

**Fig. 2:**
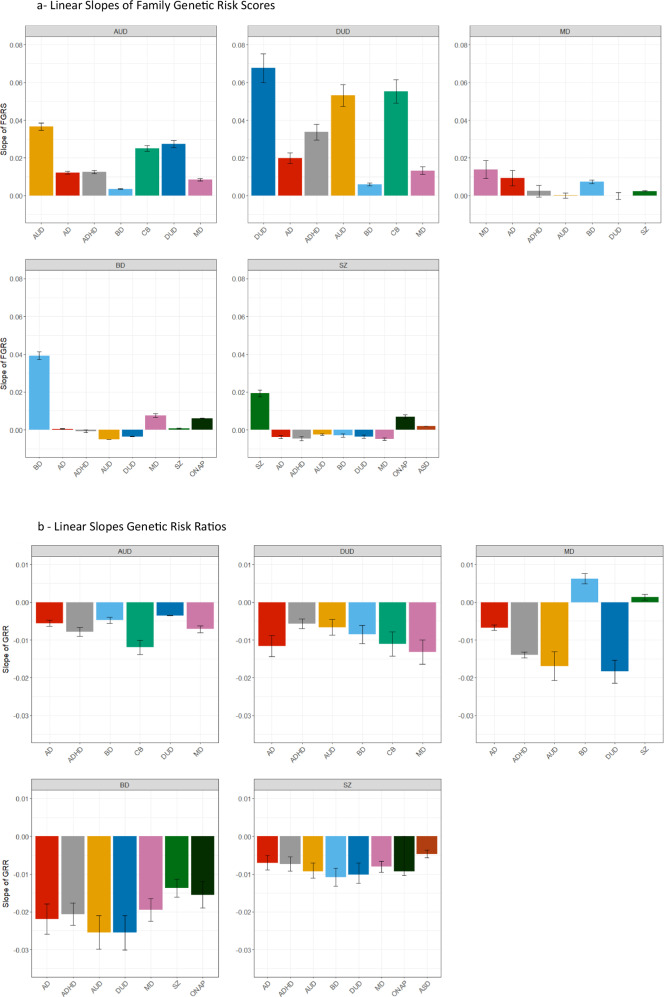
Linear Slopes of Family Genetic Risk Scores and Genetic Risk Ratios for Alcohol Use Disorder, Drug Use Disorder, Major Depression, Bipolar Disorder and Schizophrenia. **a** The linear component of the slope of the Family Genetic Risk Scores across all number of episodes for all secondary disorders for, respectively, Drug Use Disorder (DUD), Alcohol Use Disorder (AUD), Major Depression (MD), Bipolar Disorder (BD), and Schizophrenia (SZ). **b** The linear component of the slope of the Genetic Risk Ratios overall number of episodes for all secondary disorders for, respectively, Drug Use Disorder (DUD), Alcohol Use Disorder (AUD), Major Depression (MD), Bipolar Disorder (BD), and Schizophrenia (SZ).

Figure 1ai contains 7 colored lines, the orange one of which represents the mean FGRS for the primary disorder AUD, and the other six the mean FGRS for the six secondary disorders, all measured *in cases of AUD as a function of their NoEs*. All of the seven lines increase with increasing NoEs but, as seen in Fig. 2a, their slopes differ considerably, being steepest for FGRS_AUD_ followed by FGRS_DUD_ and FRGS_CB_ and flattest for FGRS_BD_.

Figure 1aii contains 6 curves of GRRs calculated from secondary disorder results presented in Fig. 1ai. Taking the green curve for CB at the top of Fig. 1aii as an example, with 4 episodes of AUD, the FGRS for CB and AUD had values of, respectively, 0.54 and 0.58, so the GRR for CB at the number of episodes equals 0.54/0.58 = 0.93. All the other curves of GRRs seen in 1aii are derived in a similar way with the numerator and denominator for each NoE equal to, respectively, the FGRS for that disorder and the denominator the FGRS_AUD_.

Overall, the 6 GRR curves in Fig. 1aii all decline at different rates as seen in Fig. 2b, steepest for CB and slowest for DUD. That is, relative to their level of genetic risk for AUD, the level of genetic risk for all these 7 disorders in AUD cases decreases with an increasing number of AUD episodes.

The pattern of results for DUD, seen in Figs. 1bi and bii, closely resemble that found for AUD, although the slope of the FGRS changes with NoEs are generally steeper (Fig. 2a). In Fig. 1bi, the upward slope for the FGRS with an increasing number of episodes for DUD is greater than that seen for any of the other secondary disorders. Therefore, in Fig. 1bii, the GRRs for these secondary disorders all decrease with increasing NoEs (Fig. 2b).

The pattern of FGRSs in cases of MD seen in Fig. 1ci differs in two ways from that seen for AUD and DUD. The FGRS curves are much more variable, with the genetic risk for MD, AD, ADHD, AUD, and DUD all rising to a maximum at between 9 and 13 MD episodes and then declining again. By contrast, the FGRS_SZ_ and FGRS_BD_ curves slightly and substantially increase, respectively, with a rising number of episodes. Examining the GRRs with MD (Fig. 1cii), the curves for DUD, AUD, and AD fall rapidly with increasing NoEs while the GRR for ADHD declines at a slower rate. By contrast, with more episodes, the GRR for SZ increases slightly and BD substantially.

The pattern for BD (Fig. 1di) is also relatively unique with the FGRS_BD_ increasingly sharply and monotonically with rising NoEs while the risks for all the secondary disorders change only slightly, some up (MD) and some down (AUD). As expected, in Fig. 1dii, the GRRs for all the secondary disorders decline with increasing NoEs, many of them substantially.

Finally, the results for SZ in Fig. 1ei show a rather substantial and monotonic risk for the FGRS_SZ_ with increasing NoEs with a much more modest rise for FGRS_ONAP_ and FGRS_ASD_. Genetic risk for all the other disorders declines with increasing NoEs. In Figs. 1eii and 2b, as is seen with BD, the GRRs for all the secondary disorders decline with increasing NoEs.

We then examine the degree of resemblance for our GRR profiles with increasing NoEs for our five primary disorders separately in men and women (Fig. 2a–e). In general, the overall pattern of GRRs were similar with the results for MD being the most discrepant (Fig. 2ci, ii). The GRRs for AUD, DUD all more quickly with increasing NoEs in males then in females. The rising slope of the GRR for BD with increasing NoEs was modestly steeper in females than males. For BD, the GRRs for most of the disorders fell more steeply with increasing NoEs in females than in males.

## Discussion

We sought, in these analyses, to determine the degree to which the NoEs for five major psychiatric and substance use disorders indexed genetic risk and reflected patterns of genetic heterogeneity. Two steps were required. First, we examined how the genetic risk for the primary disorder and secondary psychiatric and substance use disorders in cases with the primary disorder changed with increasing NoEs. Second, we explored how the genetic risk for secondary disorders changed in magnitude relative to the genetic risk for the primary disorder with increasing NoEs—what we here call the GRR.

From the rich set of findings in this study, we emphasize five. First, for both AUD and DUD, the pattern of findings was relatively simple and similar. Both genetic risk for the primary disorder and all the secondary disorders increased monotonically with increasing NoEs. That genetic risk would rise with increasing NoEs for the primary disorders is consistent with prior studies [8, 12, 14]. While we have no proven explanation for this finding, one plausible one would begin with the common-sense assumption that an episode of illness occurs when the total liability—from both genetic environmental sources—exceeds some threshold. The higher the genetic risk, the smaller the additional environmental stressor needs to be to precipitate a recurrence. So, across a lifespan, those with higher risk would more often confront environmental stressors of sufficient magnitude to precipitate a relapse. Importantly, for AUD and DUD, the genetic risk for the primary disorder increased with increased NoEs faster than the genetic risks for all the secondary disorders. So, the GRRs for the secondary disorders all fell with an increasing number of episodes. This means that the genetic profile for AUD and DUD changed with increasing recurrences. The primary genetic risk got progressively stronger, and the genetic risk became purer as the contribution from the genetic risk to the secondary disorders became progressively weaker.

**Fig. 3:**
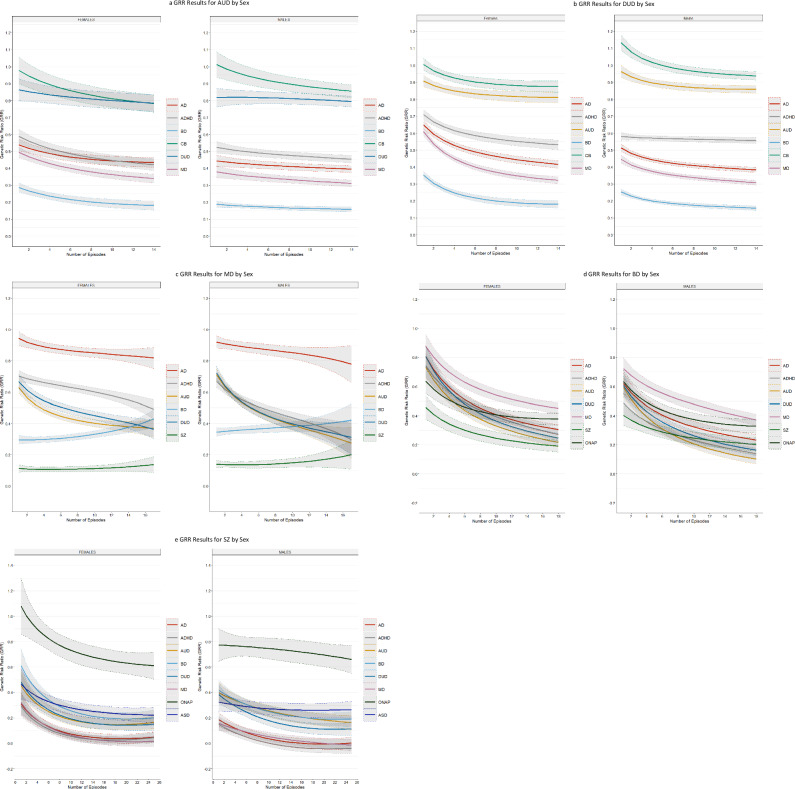
Genetic Risk Ratios by Sex for Alcohol Use Disorder, Drug Use Disorder, Major Depression, Bipolar Disorder and Schizophrenia. **a** Genetic Risk Ratios in Males and Females with Alcohol Use Disorder for number of episodes from 1 to 14 for all the secondary disorders/traits listed in the legend for Fig. 1a. **b** Genetic Risk Ratios in Males and Females with Drug Use Disorder for number of episodes from 1 to 14 for all the secondary disorders/traits listed in the legend for Fig. 1b. **c** Genetic Risk Ratios in Males and Females with Major Depression for number of episodes from 1 to 16 for all the secondary disorders/traits listed in the legend for Fig. 1c. **d** Genetic Risk Ratios in Males and Females with Bipolar Disorder for number of episodes from 1 to 18 for all the secondary disorders/traits listed in the legend for Fig. 1d. **e** Genetic Risk Ratios in Males and Females with Schizophrenia for number of episodes from 1 to 25 for all the secondary disorders/traits listed in the legend for Fig. 1e.

Second, the picture for MD was different (see Fig. 3). Consistent with prior studies [10], higher FGRS_MD_ was associated with a higher NoEs but the relationship was curvilinear maximizing at 12 episodes and then modestly declined. A similar pattern was seen for the genetic risk for AD, ADHD, DUD, and AUD. Furthermore, the FGRS for AUD and DUD barely rose with a modest number of episodes and then declined appreciably. Uniquely in these analyses, the genetic risk for two secondary disorders – SZ and especially BD increased at a faster rate than did that of MD with increasing NoEs. This produced a complex picture when we examined the GRRs. With increasing NoEs, the level of genetic risk in MD probands for AD decreased modestly, ADHD moderately and AUD and DUD rather quickly. By contrast, the genetic risk for BD and SZ in MD cases increased with increasing NoEs with the effect largely impacting on those with 12 or greater episodes. These results suggest that affected individuals with few episodes of MD will have, on average, high genetic risks for ADHD, AUD, and DUD while those with highly recurrent MD will have lower levels of these genetic risk but have a considerably higher risk for BD.

Third, the pattern of results for BD is also relatively unique. Genetic risk for BD rose sharply as a function of increasing NoEs compared to all the secondary disorders which showed a mixture of modest increases and decreases. In GRR analyses, the level for all the secondary disorders declined substantially with greater NoEs. These results suggest that sampling BD cases with high rates of recurrence would produce a sample not only with a high overall genetic risk, but with a relatively pure genetic signal as the genetic contributions of all other secondary disorders, including MD, minimized.

Fourth, in SZ, we see a distinction between our standard group of secondary disorders (AD, ADHD, AUD, BD, DUD, and MD) whose FGRS declined with increasing NoEs and two additional disorders added a priori to this analysis (ONAP and ASD) where their FGRS increased modestly. This is reflected in the GRR analyses where we saw a rapid decline in the standard secondary disorders with increasing recurrence rates of SZ and a slower decline of ONAP and ASD. As with BD, our results suggest that studying highly recurrent cases of SZ would result in a strong and relatively pure genetic signal with the exception of a substantial genetic risk to the closely related syndrome of ONAP.

Fifth, it is of interest to compare our results for MD and BD. The most striking difference between these disorders was that in BD, the GRR for MD declined with increases NoEs while in MD, the GRR of BD increased with increasing NoEs. Rephrased, this suggests that in BD, a genetic risk for MD is correlated with a low NoEs while in MD, a genetic risk for BD predicts a high NoEs. Also, FGRS_BD_ was considerably more strongly related to NoEs than was FGRS_MD_, suggested that high recurrence rates are more strongly “encoded” in the genetic liability to BD than MD.

## Limitations

These results should be interpreted in the context of six potential methodological limitations. First, the validity of our findings is dependent upon the quality of the diagnoses in the Swedish registries. The validities of the hospital diagnoses for SZ and BD in Sweden are well supported [21–23] as are the validity of MD on the basis of its prevalence, sex ratio, sibling and twin correlations and associations with known psychosocial risk factors [24, 25]. The validities of AUD and DUD are supported both by the high rates of concordance across ascertainment methods [26, 27] and the patterns of resemblance in relatives similar to those found in personally interviewed samples [28, 29]. The diagnosis of ADHD in Sweden is validated by its close relationship with the receipt of stimulant medication [30].

Second, the power of the FGRS as a measure of genetic risk is based on the availability, for Swedish born individuals residing in Sweden, of extensive high quality phenotypic data on large numbers of close and extended relatives. Therefore, this statistic is best applied to populations with registry information similar to that of Sweden. Furthermore, the FGRS differs qualitatively from polygenic risk scores in that it obtains genetic risk indirectly from rates of illness in relatives and not from DNA sequence. Of note, recent analyses in and simulations based on the Danish registry have shown that, correcting for the substantial error variance in both statistics, the genetic signal obtained from a polygenic risk scores and from an FGRS-like statistics are highly correlated [31].

Third, we do not have precise estimates of beginning and end of disease episodes and have to estimate them for registrations which likely introduces some error.

Fourth, in addition to exploring the stability of our findings across sex, we also examined their stability across two birth cohorts (appendix Fig. 2a–e): 1955–1964 and 1965–1974. While a substantial similarity in overall pattern was observed, some meaningful differences were detected. We saw higher GRRs for CB and MD in the more recent cohort for AUD and DUD cases. In MD, GRRs for AD were higher in the more recent cohort which also displayed a less robust rise in the GRR for BD with increasing NoEs. For BD and SZ, GRRs were generally higher in the more recent cohort, though the overall pattern was similar. Some of these changes could arise from alterations in the diagnosis system (ICD-10 was introduced to Sweden in 1997), the functioning of the Swedish registries (e.g. primary care data largely became available after 2000) and/or changes in prevalence (e.g. rising rates of diagnosed ADHD and DUD in Sweden over recent decades [32, 33]).

Fifth, to address the temporal stability of our findings for NoEs, we examined the GRR of recurrence rates 0–5 and 6–10 years after onset in our primary disorders (appendix Fig. 3a–e). Consistent with prior findings of a stable pattern of recurrence across the lifespan in MD and BD [34], the GRR patterns are relatively stable over these two 5 year periods.

Finally, our main analyses did not consider the impact of comorbidities in our primary disorders on our results. Therefore, we explored the impact, on the GRR profiles for our primary disorders, of censoring, from our samples, individuals with particular patterns of comorbidity (appendix Fig. 4a–e). Overall, the effects were modest and typically impacted on the GRR only for the eliminated comorbid disorder. These results suggest that our overall findings are not largely the result of patterns of comorbidity of our primary disorders.

## Conclusions

The level of recurrence for a representative set of psychiatric and substance use disorders is both a useful measure of genetic risk and of potential genetic heterogeneity. In most current psychiatric studies that utilizes genetic factors, the main focus is solely on diagnostic status. Our results suggest that this approach is inefficient, as a measure as simple to obtain as number of episodes provides substantial additional genetic information.

New aspects of comorbidity are revealed when they are examined from the perspective of NoEs. For example, individuals with MD with few recurrences have relatively high genetic risk for ADHD, AUD, and DUD but low genetic risk for BD. Those with high levels of recurrence have the opposite pattern. Patients with few episodes of BD have a considerably higher genetic risk for SZ than those with many episodes and in patients with SZ the level of genetic risk for BD declines with increasing NoEs.

Our findings also have implication for patient selection for genetic studies. For four of our five disorders—AUD, DUD, BD, and SZ,—our results suggest that selectively studying patients with high levels of recurrence would bring a double benefit—higher overall genetic risk for the primary disorder and reduced genetic heterogeneity.

Finally, our findings challenge the hypothesis that diagnostic categories capture much of the critical genetic variability that we seek to understand in psychiatric disorders. Rather, they suggest that beneath diagnostic categories are further assessable sources of important variation that can add appreciable new information about the patterns of genetic risk.

## Supplementary information

Appendix
